# Neuron‐specific deletion of presenilin enhancer2 causes progressive astrogliosis and age‐related neurodegeneration in the cortex independent of the Notch signaling

**DOI:** 10.1111/cns.13454

**Published:** 2020-09-22

**Authors:** Hui‐Ru Bi, Cui‐Hua Zhou, Yi‐Zhi Zhang, Xu‐Dong Cai, Mu‐Huo Ji, Jian‐Jun Yang, Gui‐Quan Chen, Yi‐Min Hu

**Affiliations:** ^1^ Model Animal Research Center, MOE Key Laboratory of Model Animal for Disease Study, Medical School Nanjing University Nanjing China; ^2^ Department of Anesthesiology The Second Affiliated Changzhou People’s Hospital of Nanjing Medical University Changzhou China; ^3^ Department of Anesthesiology, Pain and Perioperative Medicine The First Affiliated Hospital of Zhengzhou University Zhengzhou China

**Keywords:** Alzheimer disease, astrogliosis, microgliosis, neurodegeneration, presenilin enhancer2

## Abstract

**Introduction:**

Presenilin enhancer2 (Pen‐2) is an essential subunit of γ‐secretase, which is a key protease responsible for the cleavage of amyloid precursor protein (APP) and Notch. Mutations on Pen‐2 cause familial Alzheimer disease (AD). However, it remains unknown whether Pen‐2 regulates neuronal survival and neuroinflammation in the adult brain.

**Methods:**

Forebrain neuron‐specific *Pen‐2* conditional knockout (*Pen‐2* cKO) mice were generated for this study. *Pen‐2* cKO mice expressing Notch1 intracellular domain (NICD) conditionally in cortical neurons were also generated.

**Results:**

Loss of Pen‐2 causes astrogliosis followed by age‐dependent cortical atrophy and neuronal loss. Loss of Pen‐2 results in microgliosis and enhanced inflammatory responses in the cortex. Expression of NICD in *Pen‐2* cKO cortices ameliorates neither neurodegeneration nor neuroinflammation.

**Conclusions:**

Pen‐2 is required for neuronal survival in the adult cerebral cortex. The Notch signaling may not be involved in neurodegeneration caused by loss of Pen‐2.

## Graphical abstract

This study reports a novel mouse model, which exhibits age‐related neurodegeneration. Forebrain neuron‐specific deletion of Pen‐2 leads to early astrogliosis followed by age‐dependent neuronal loss and severe inflammatory responses in the adult cerebral cortex. The Notch signaling does not play a critical role in Pen‐2‐dependent neuronal survival.

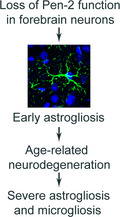

**Highlights**

- Conditional deletion of Pen‐2 in the forebrain causes age‐related cortical atrophy in mice.
- Deletion of Pen‐2 leads to early astrogliosis in the adult cortex.
- Deletion of Pen‐2 results in age‐related neurodegeneration and neuroinflammation.
- The expression of NICD rescues neither neuronal loss nor inflammatory responses in the cortex.

## 1 INTRODUCTION

Alzheimer disease (AD) is the most common form of dementia and has become a global problem for the elderly. AD is clinically characterized by progressive cognitive deficits and morphologically by neuronal loss and the presence of amyloid plaques and neurofibrillary tangles.^1^, ^2^ Based on the start time of symptoms, AD can be categorized into two subtypes, early‐onset, and late‐onset. Major genetic causes for early‐onset of AD include mutations on *APP* (amyloid precursor protein), *Psen1* (presenilin1), and *Psen2* (presenilin2).^3^, ^4^, ^5^ These mutations cause familial AD that displays clinical symptoms in midlife.^1^ It is believed that Aβ accumulation,^6^ tau hyperphosphorylation,^7^ loss of presenilin function,^8^, ^9^ metabolic dysfunction,^10^, ^11^ and epigenetic abnormality^12^, ^13^ play critical roles in the pathogenesis of AD. So far, there are still no validated prognostic or diagnostic tests.^14^

γ‐Secretase is a well‐known protease responsible for the intramembranous cleavage of APP and Notch to produce Aβ and Notch intracellular domain (NICD).^15^, ^16^ It contains four essential subunits including presenilin, presenilin enhancer2 (Pen‐2), nicastrin, and anterior pharynx defective1 (Aph‐1).^17^ The *Pen‐2* gene, located on chromosome 19q13, is composed of 4 exons.^18^ It has been reported that downregulation of Pen‐2 is associated with reduced levels of presenilin and impaired maturation of nicastrin.^19^ Interestingly, production of N‐ and C‐terminal fragments of presenilin1 is abolished by downregulation of Pen‐2 and is enhanced by overexpression of Pen‐2.^20^ Therefore, Pen‐2 is required for the endoproteolysis of presenilin. Although previous evidence has demonstrated that Pen‐2 may protect zebrafish embryos from apoptosis,^21^, ^22^ physiological functions of Pen‐2 in the adult brain remain largely unknown due to early embryonic lethality in germ‐line *Pen‐2* knockout mice.^23^ Recently, we employed conditional knockout (cKO) techniques to overcome the lethality problem caused by germ‐line deletion of Pen‐2. Functional analyses on neural progenitor cells (NPCs)‐specific *Pen‐2* KO mice reveal that Pen‐2 controls the fate switch of intermediate progenitors from radial glial progenitors in the developing cortex through a Notch‐dependent mechanism,^24^ suggesting that Pen‐2 is critical for the maintenance of neural stem cells.

Recent evidence has shown that mutations on *Pen‐2* are associated with familial AD.^25^, ^26^, ^27^ Thus, Pen‐2 is involved in the pathogenesis of AD. However, it remains unknown whether Pen‐2 regulates neuronal survival in the adult cerebral cortex. To address this question, we generated forebrain neuron‐specific *Pen‐2* cKO mice by crossing floxed *Pen‐2* ^24^ with a transgenic mouse expressing Cre under the promoter of α‐calcium‐calmodulin‐dependent kinase II (*CaMKIIα*).^28^, ^29^, ^30^ We show that loss of Pen‐2 leads to early astrogliosis, age‐related reduction on the cortical size, and age‐dependent neurodegeneration. We observe enhanced inflammatory responses in *Pen‐2* cKO mice. Moreover, we find that expression of NICD does not improve neurodegenerative phenotypes in *Pen‐2* cKO mice. Overall, this study highlights an essential role of Pen‐2 in neuronal survival in the adult cortex.

## 2 MATERIALS AND METHODS

### 2.1 Animals

We employed a strategy similar to those previously reported ^31^, ^32^ to generate forebrain neuron‐specific *Pen‐2* cKO mice for this study. We bred *Pen‐2^f/+^* ^24^ with the *CaMKIIα‐Cre* mouse ^29^, ^30^ to obtain *Pen‐2^f/+^;CaMKIIα‐Cre*, which was then bred to *Pen‐2^f/f^* to produce *Pen‐2^f/f^* (control), *Pen‐2^f/+^;CaMKIIα‐Cre* (control), and *Pen‐2^f/f^;CaMKIIα‐Cre* (*Pen‐2* cKO) mice. To generate *Pen‐2* cKO animals expressing NICD in the forebrain, *Pen‐2^f/+^;CaMKIIα‐Cre* mice were crossed to *Pen‐2^f/f^; LSL‐N1ICD* to produce *Pen‐2^f/+^; CaMKIIα‐Cre;LSL‐N1ICD* (control), *Pen‐2^f/f^; CaMKIIα‐Cre* (*Pen‐2* cKO) and *Pen‐2^f/f^;CaMKIIα‐Cre;LSL‐N1ICD* (*Pen‐2* cKO;*N1ICD*). Tail DNAs were used for genotyping by PCR. To detect the floxed *Pen‐2* allele, the following primers were used: GACCCGTAGAAGAGCAGTCAGT (forward) and ATAAAGAATAGGCTGGGTGGTG (reverse). To detect *N1ICD* gene, the following primers were used: AAGTGCAGGTGCCAGAACAT (forward) and GCAGCATCTGAACGAGAGTA (reverse).

The genetic background of the mice was C57BL/6, and both genders were used in this study. The mice were group‐housed (4‐5 per cage) and had free access to food and water. They were maintained in an SPF room in the core facility of the Model Animal Research Center (MARC) at Nanjing University. The animal room was maintained under constant humidity and temperature (25 ± 1°C). The light‐cycle of the animal room was automatically controlled. Mouse breeding was conducted under an animal protocol approved by the IACUC in the MARC of Nanjing University. All the experiments were performed in accordance with the Guide for the Care and Use of Laboratory Animals of Nanjing University.

### 2.2 Nissl staining and measurement on the thickness of the cortex

Mice at 2, 3, 6, or 9 months were euthanized by CO_2_ and were then perfused with PBS. The brain was fixed in 4% paraformaldehyde (PFA) overnight at 4°C, followed by dehydration using ethanol. After being embedded in paraffin, four brains were placed into one block, which was sectioned sagittally at the thickness of 10 μm using a microtome (Leica Microsystems, Bannockburn, IL, United States). A total of 5 sagittal brain sections, spaced at 300 μm apart, were used for Nissl staining. Sections were deparaffinized in xylene and rehydrated in ethanol. Sections were treated with 0.1% cresyl‐violet for 1 minute and were then washed with distilled water. Dried sections were sealed using neutral resin (Sinopharm Chemical Reagent Co. Ltd., Shanghai). We used a method described by Acx and colleagues (2017) to measure the thickness of the cortex. First, Nissl‐stained images were captured using an Olympus BX53 microscope. Second, the thickness of the parietal‐occipital cortex overlying the hippocampus was measured and averaged for mice at different ages (3 mice per group per age).

### 2.3 Immunohistochemistry (IHC)

We used a method described recently.^33^, ^34^ Sections were first deparaffinized and were then rehydrated using ethanol, boiled in 0.01 mol/L sodium citrate buffer (pH = 6.0) for 25 minutes. Sections were blocked by PBS containing 5% bovine serum albumin (BSA) for 30 minutes and incubated overnight at 4°C with the following antibodies: anti‐NeuN (ABN781:1000; Millipore), anti‐MAP2 (MAB3418, 1:500; Millipore), anti‐GFAP (A14673; 1:500; Abclonal), and anti‐Iba1 (019‐19741; 1:500; Wako). On the next day, the sections were incubated for 1 hour at room temperature in PBS with secondary antibodies (1:500) including Alexa Fluor 488 goat anti‐mouse/anti‐rabbit and Alexa Fluor 594 goat anti‐mouse/anti‐rabbit (Invitrogen). Images were captured and analyzed using a ZEISS LSM‐880 confocal laser‐scanning microscope.

### 2.4 Cell counting

We used a method described recently by Acx and colleagues (2017). Briefly, three sagittal sections spaced 300 μm apart were used for IHC and at least three mice were included for each genotype at each age. For counting of NeuN‐positive (NeuN^+^), GFAP^+^ or Iba1^+^ cells in the cortex, IHC images for the parietal‐occipital cortex overlying the hippocampus morphologically were captured under the 10 × objective lens of an Olympus BX53 microscope. ImageJ was used to count NeuN^+^, GFAP^+^, or Iba1^+^ cells in each image. The number of cells in each image (876 × 660 μm^2^) was then averaged across 3 sections.

### 2.5 Tissue preparations for Western analysis

Mice were euthanized by CO_2_.^35^, ^36^ Tissues for different brain regions were freshly collected from mice at 2, 3, 6, or 9 months and were quickly placed into liquid nitrogen. Samples were stored at −80°C until use. Tissues from the cortex were homogenized in cold radio immunoprecipitation assay (RIPA) lysis buffer (1% NP40, 0.1% SDS, 0.5% sodium deoxycholate and 1 mmol/L EDTA in TBS) containing protease and phosphatase inhibitors (Thermo) and were then centrifugated at 12 000 *g* for 15 minutes. The RIPA‐soluble supernatants were used as total protein lysates. Protein concentrations were analyzed using a BSA method described previously.^24^, ^32^ Normalized cortical protein lysates with a total of 40 μg protein were separated on a 10% SDS–PAGE (Invitrogen) and then transferred to nitrocellulose membrane. The latter was blocked with 5% dry milk for 1 hour and then incubated with primary antibodies overnight. The membrane was washed with TBS for three times and then reacted with a Li‐Cor IRDye infrared dye‐coupled secondary antibody. Membranes were scanned, and data were analyzed using the Odyssey Infrared Imaging System (Li‐Cor). The following primary antibodies were used: anti‐Pen‐2 (A15172; 1:1000; Abclonal), anti‐nicastrin (34‐9200; 1:500; Invitrogen), anti‐APP (A8717; 1:1000; Sigma‐Aldrich), anti‐Iba1 (382207; 1:1000; ZENBIO), anti‐GFAP (A14673; 1:1000; Abclonal), anti‐GPADH (CW0100; 1:10,000; CWBIO), and anti‐β‐actin (CW0096; 1:10,000, CWBIO).

### 2.6 RNA Extraction and quantitative real‐time PCR

Total RNAs from mouse cortices were purified using the TRIzol reagent (Invitrogen) according to the manufacturer's instructions. RNA integrity was confirmed by the detection of 28S and 18S rRNA bands in agarose gel electrophoresis. RNA concentration was measured using a Nanodrop spectrophotometer. Equal amounts (1 μg) of RNAs were reverse transcribed into cDNA using PrimeScript RT reagent Kit (Takara). Quantitative real‐time PCR (qPCR) was performed by the ABI StepOne Plus system. *Gapdh* was used as the internal control in each PCR reaction. The PCR reactions were performed three times independently, and each sample was loaded in duplicates. The average CT values were used to calculate relative levels. The primer sequences used were as follows. For *Pen‐2*: TGGATTTGCGTTCCTGCCTTTTCT (forward) and ATGAAGTTGTTAGGGAGTGCC (reverse). For *GFAP*: AGGTGG AGAGGGACAACTTT (forward) and TCTGCCTCCTGTCTATACGC (reverse). For *Iba1*: GCTTTTGGACTGCTGAAGGC (forward) and GTTTGGACGGCAGATCC TCA (reverse). For *IL1β*: GCAACTGTTCCTGAACTCAACT (forward) and ATCT TTTGGGGTCCGTCAACT (reverse). For *TNFα*: CCTCCCTCTCATCAGTTCTAT GG (forward) and GGCTACAGGCTTGTCACTCG (reverse). For *N1ICD*: TGTCA ATGTTCGAGGACCAG (forward) and GCAGCATCTGAACGAGAGTA (reverse). For *Gapdh*: AATGTGTCCGTCGTGGATCT (forward) and CCCTGTTGCTGTAG CCGTAT (reverse).

### 2.7 Statistical Analysis

Data were presented as the mean ± the standard error of the mean (SEM). All the data were subject to Normality test using the Shapiro‐Wilk method in SPSS. While three sets of data including Western blotting on nicastrin (for *Pen‐2* cKO) and qPCR analyses on *IL1β* (for *Pen‐2* cKO at 6 months) and *NICD* (for control) did not exhibit normal distribution (*P* < 0.05), the remaining showed normal distribution (*P* > 0.05). For data without normal distribution, *P* values were obtained from Univariate Analysis of Variance (ANOVA), by which we analyzed genotype effects between different groups. For data with normal distribution, Student *t* test (two‐tailed, unpaired) was conducted to analyze genotype effects. *P* < 0.05 was considered statistically significant and *P* < 0.01 highly significant.

## 3 RESULTS

### 3.1 Generation of forebrain neuron‐specific *Pen‐2* cKO mice

**Figure 1:**
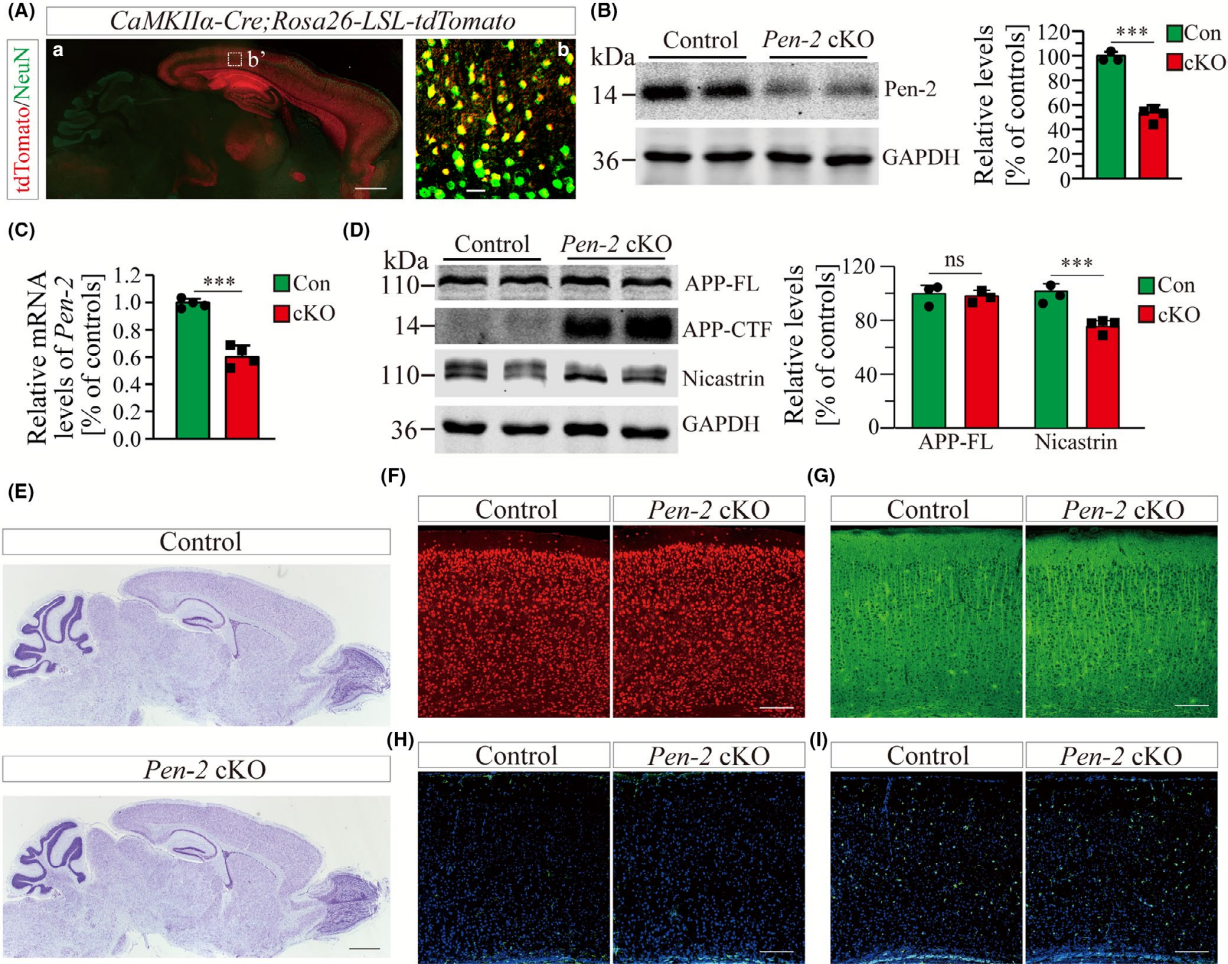
Molecular characterization of forebrain neuron‐specific *Pen‐2* cKO mice. A, Co‐staining for NeuN and tdTomato. *CaMKIIα‐Cre;Rosa26‐LSL‐tdTomato* mice were used to collect brain sections. There was wide expression of tdTomato in the cortex, the hippocampus, the olfactory bulb, and the striatum but not the cerebellum. Most NeuN^+^ cells were positive for tdTomato. The boxed area in (a) was enlarged as (b). Scale bar is 500 μm in (a) but 25 μm in (b). B, Western analysis on Pen‐2. Cortical protein samples for control (Con) and *Pen‐2* cKO (cKO) mice at 3 mo were used. There was significant difference on Pen‐2 levels between control and *Pen‐2* cKO mice (***P < 0.001; n = 3‐4 mice per group). C, Relative *Pen‐2* mRNA levels. There was significant difference between control (Con) and *Pen‐2* cKO (cKO) mice (***P < 0.001; n = 4 per group). D, Western analyses for APP‐FL, APP‐CTF and nicastrin. Cortical samples at 3 mo were used. There was no significant difference on protein levels of APP‐FL between control (Con) and *Pen‐2* cKO (cKO) mice (*P* > 0.6; n = 3 per group). There was significant difference on protein levels of nicastrin between control and *Pen‐2* cKO mice (***P < 0.001; n = 3‐4 per group). E, Nissl staining. There was comparable brain size in control and *Pen‐2* cKO mice at 2 mo. Scale bar is 1 mm. F‐I, Representative fluorescence IHC images for NeuN (F), MAP2 (G), GFAP (H) and Iba1 (I). Images were taken from cortices of control and Pen‐2 cKO mice at 2 mo. Scale bar is 200 μm

*Pen‐2^f/f^* ^24^ mice were used to generate *Pen‐2* cKO mice in which Cre is expressed in excitatory neurons in the postnatal forebrain. To visualize the expression pattern of Cre, we crossed a *tdTomato* reporter line to the *CaMKIIα‐Cre* mouse. Abundant NeuN^+^/tdTomato^+^ cells were observed specifically in the cortex and the hippocampus of *CaMKIIα‐Cre;LSL‐tdTomato* mice (Figure 1A). To quantify the inactivation efficiency of Pen‐2, we analyzed cortical samples from *Pen‐2* cKO mice at 3 months. First, Western analysis showed a significant reduction on protein levels of Pen‐2 in mutants compared with controls (Figure 1B and Figure (S1)). Second, qPCR analysis revealed significantly decreased levels of *Pen‐2* mRNAs in mutants (Figure 1C). The residual amount of Pen‐2 in *Pen‐2* cKO cortices likely came from glia, interneurons, and some neurons in which Cre was not expressed (Figure 1A,B). As expected, conditional deletion of Pen‐2 did not affect the expression of APP in postnatal neurons in the cortex (Figure 1D and Figure S1). We observed massive accumulation of the C‐terminal fragment of APP (APP‐CTF) (control = 100 ± 6%; cKO = 3812 ± 183%; n = 3 mice per group; *P* < 0.001) and significant reduction on nicastrin protein in *Pen‐2* cKO cortical samples compared with controls (Figure 1D and Figure S1). Overall, loss of Pen‐2 significantly impaired γ‐secretase activity in the brain.

To find out whether conditional inactivation of Pen‐2 affected the general morphology of the cortex, we conducted Nissl staining using brain sections from mice at 2 months. We found that the architecture of the cortex and the hippocampus was comparable between control and *Pen‐2* cKO mice (Figure 1E). Moreover, IHC experiments revealed no detectable change on the immuno‐reactivity of NeuN, MAP2, GFAP, or Iba1 in *Pen‐2* cKO mice compared with controls (Figure 1F‐I). Together, cortical development in general was unaffected by deletion of Pen‐2.

### 3.2 Age‐dependent cortical atrophy and neuronal loss in *Pen‐2* cKO mice

**Figure 2:**
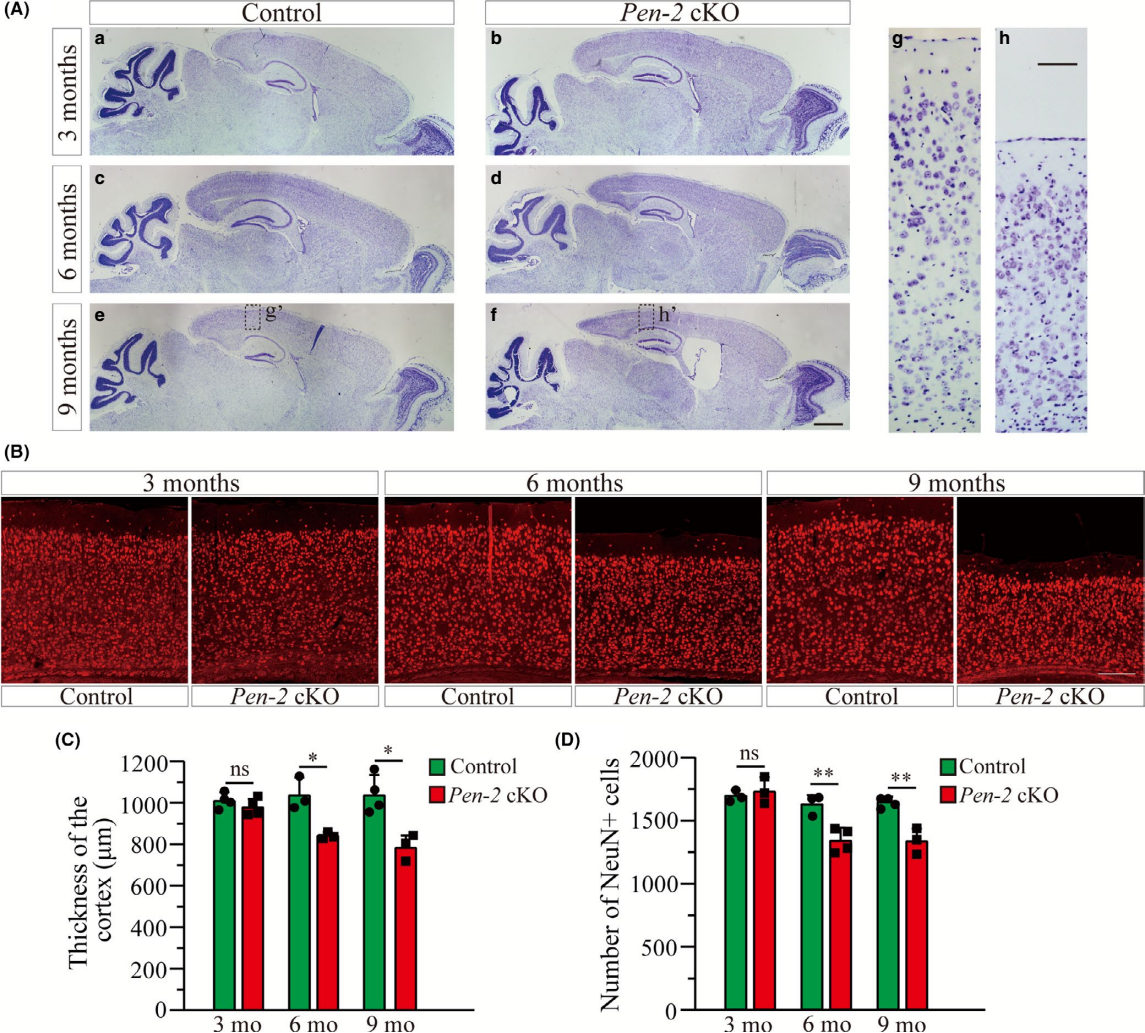
Age‐dependent neurodegeneration in *Pen‐2* cKO mice. A, Nissl staining. Brain sections of mice at 3, 6, and 9 mo were used. There was cortical atrophy and enlarged lateral ventricle in *Pen‐2* cKO mice at 6 and 9 mo compared with controls. Boxed areas in (e) or (f) were enlarged as (g) and (h), respectively. Scale bar is 1 mm for (a‐f) or 100 μm for (g‐h). B. Representative fluorescence images for NeuN IHC taken from cortices. Brain sections from mice at 3, 6, and 9 mo were used. Scale bar is 200 μm. C, Cortical thickness. There was no significant difference on the size of the cortex between control and *Pen‐2* cKO mice at 3 mo. There was significant decrease in *Pen‐2* cKO mice at 6 (*P < 0.05; n = 3 mice per group) or 9 mo (*P < 0.05; n = 3‐4 per group). D, Averaged number of NeuN^+^ cells. There were significant decreases on NeuN^+^ cells in *Pen‐2* cKO mice at 6 (**P < 0.01; n = 3‐4 per group) and 9 (**P < 0.01; n = 3‐4 per group)

To determine whether neuronal survival was affected in aged *Pen‐2* cKO mice, Nissl staining (Figure 2A) and NeuN IHC (Figure 2B) were performed using brain sections from mice at ages ranging from 3 to 9 months. First of all, Nissl staining revealed a thin cortex in *Pen‐2* cKO mice at 6 or 9 months compared with littermate controls (Figure 2A), and quantification results confirmed significantly reduced thickness of the cortex (Figure 2C). In contrast, the thickness of the cortex was not different at 3 months (Figure 2C). Secondly, NeuN IHC also showed thinner cortex in *Pen‐2* cKO mice at 6 and 9 months than in controls (Figure 2B). The number of NeuN^+^ cells was not changed in *Pen‐2* cKO mice at 3 months compared with controls, but it was significantly decreased at 6 and 9 months (Figure 2D), indicating age‐related neurodegeneration in the cortex of *Pen‐2* cKO mice.

### 3.3 Progressive astrogliosis in *Pen‐2* cKO mice

**Figure 3:**
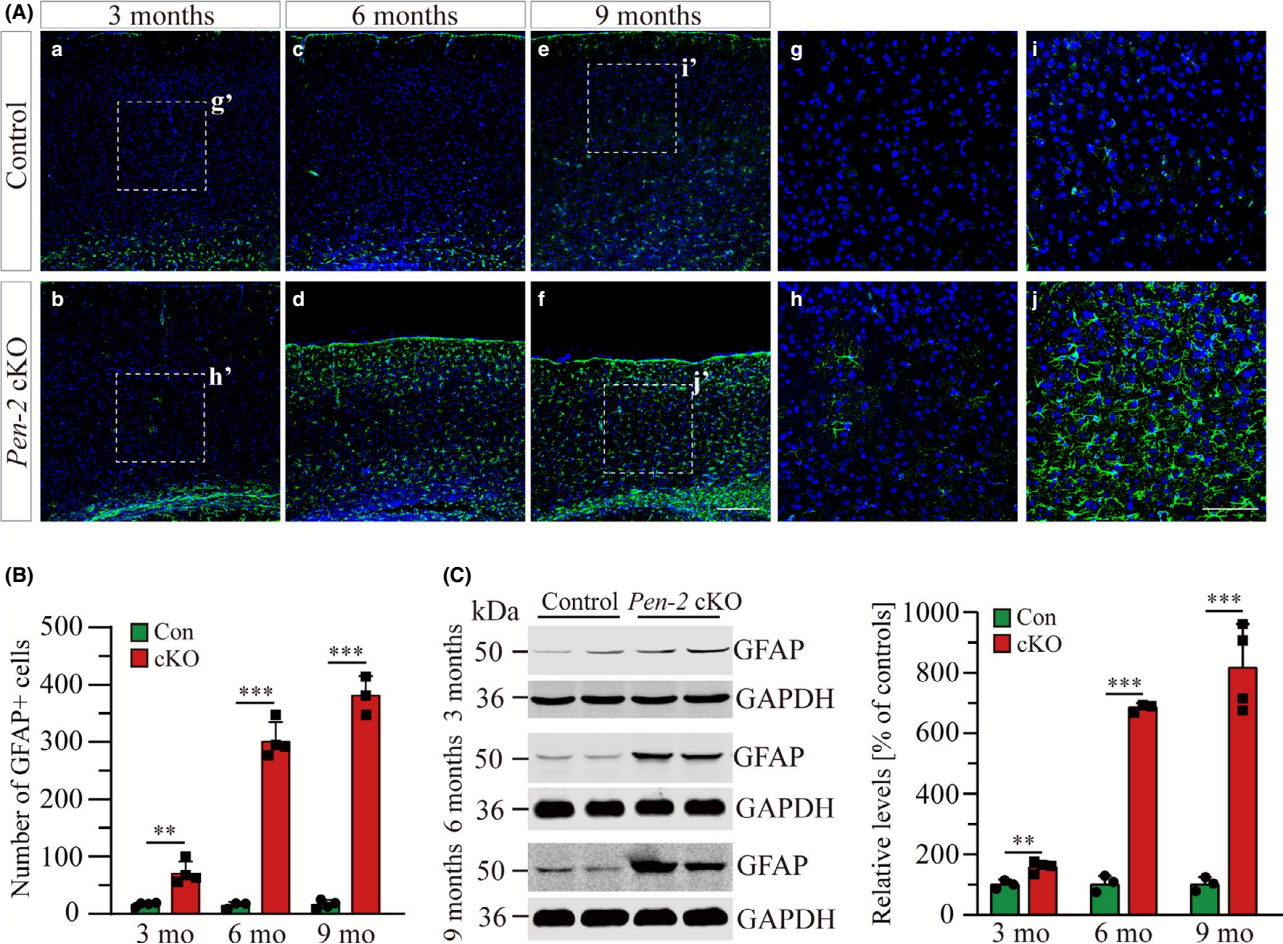
Progressive astrogliosis in *Pen‐2* cKO mice. A, Representative images for fluorescence IHC on GFAP in the cortex. Brain sections of mice at 3, 6, and 9 mo were used. The immuno‐reactivity of GFAP was increased in *Pen‐2* cKO mice since 3 mo of age compared with controls. Boxed areas in (a), (b), (e), and (f) were enlarged as (g), (h), (i), and (j), respectively. Scale bar is 200 μm for (a‐f) or 100 μm for (g‐j). B. Averaged number of GFAP^+^ cells in the cortex. There was significant difference between control (Con) and *Pen‐2* cKO (cKO) mice at each age (3 mo: **P < 0.01; 6 mo: ***P < 0.001; 9 mo: ***P < 0.001; n = 3‐4 mice per group). Analysis of variance (ANOVA) revealed significant Age (F = 115.3, *df*2/16, *P* < 0.001) and Age × Genotype effects (*F* = 116.5, *df*2/16, *P* < 0.001), suggesting progressive astroglial activation. C, Western analysis on GFAP. Cortical samples at 3, 6, and 9 mo were used. GFAP levels in *Pen‐2* cKO (cKO) mice differed from those in controls (Con) at each age (*P* < 0.05; n = 3‐4 per group). The increase in GFAP levels was above 6‐fold in *Pen‐2* cKO cortices compared with controls at 6 or 9 mo

To examine whether deletion of Pen‐2 affected astrocytes, we performed IHC on GFAP using mice at different ages (Figure 3A). While the immuno‐reactivity of GFAP was increased in the cortex of *Pen‐2* cKO mice at 3 months compared with controls, it was rigorously detected at 6 and 9 months (Figure 3A). Cell counting results showed significant increase on the number of GFAP^+^ cells in *Pen‐2* cKO cortices at each age (Figure 3B). Moreover, we conducted Western analysis on GFAP using total cortical homogenates prepared from mice at the above different ages (Figure 3C and Figure S2). While GFAP protein exhibited a small but significant increase in *Pen‐2* cKO mice at 3 months of age, it was robustly increased at 6 and 9 months (Figure 3C). Finally, we performed qPCR to examine *GFAP* mRNAs, which were significantly increased in *Pen‐2* cKO mice at 6 and 9 months compared with controls (data not shown). Thus, inactivation of Pen‐2 caused progressive astrogliosis in the cortex.

### 3.4 Age‐related microgliosis and neuroinflammatory responses in *Pen‐2* cKO mice

**Figure 4:**
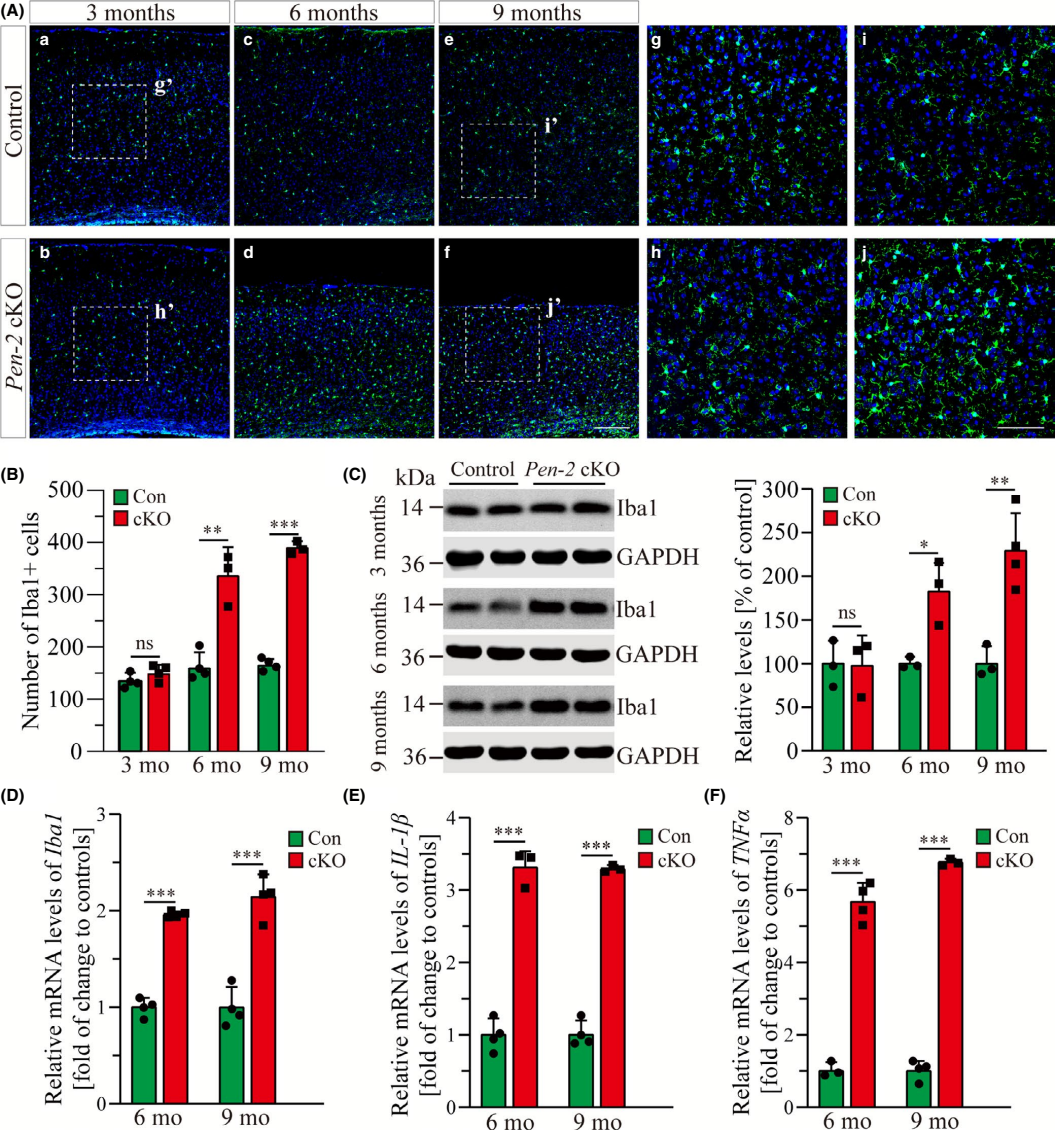
Age‐related microgliosis in *Pen‐2* cKO mice. A, Representative images for fluorescence IHC on Iba1 in the cortex. Mice at 3, 6, and 9 mo were examined. Note that the immuno‐reactivity of Iba1 was not increased in *Pen‐2* cKO mice at 3 mo compared with controls. Boxed areas in (a), (b), (e), and (f) were enlarged as (g), (h), (i), and (j), respectively. Scale bar is 200 μm for (a‐f) or 100 μm for (g‐j). B, Averaged number of Iba1^+^ cells in the cortex. There was significant difference between control (Con) and *Pen‐2* cKO (cKO) mice at each age (3 mo: *P* > 0.2; 6 mo: **P < 0.01; 9 mo: ***P < 0.001; n = 3‐4 mice per group). C, Western analysis on Iba1. Note that Iba1 levels in *Pen‐2* cKO (cKO) mice did not differ from those in controls (Con) at 3 mo of age but were increased at 6 and 9 mo (3 mo: not significant; 6 mo: *, *P* < 0.05; 9 mo: **P < 0.01; n = 3‐4 per group). D‐F, qPCR analyses for *Iba1* (D), *IL‐1β* (E), and *TNFα* (F). Data for 6 and 9 mo were presented. There was significant increase on levels for *Iba1* (D), *IL‐1β* (E), and *TNFα* (F) in *Pen‐2* cKO (cKO) mice compared with controls (Con) at either age (***P < 0.001; n = 3‐4 per group)

We next analyzed microglia by performing IHC on Iba1. First, the immuno‐reactivity of Iba1 was comparable in control and *Pen‐2* cKO cortices at 3 months, but it was increased in *Pen‐2* cKO mice at 6 and 9 months (Figure 4A). Quantification results showed that the number of Iba1^+^ cells was not changed in the cortex of *Pen‐2* cKO mice at 3 months (Figure 4B) and that it was highly increased at 6 and 9 months (Figure 4B). Thus, cell counting results were consistent with those from IHC. Next, we performed Western analysis on Iba1 (Figure 4C and Figure S3). Indeed, relative Iba1 levels were unaltered in *Pen‐2* cKO mice at 3 months (*P* > 0.4) but were increased at 6 and 9 months compared with controls (Figure 4C). Finally, we found that mRNA levels for *Iba1* were significantly increased in *Pen‐2* cKO mice at 6 or 9 months (Figure 4D). Overall, there was striking age‐related microgliosis in *Pen‐2* cKO mice.

Next, we examined expression for neuroinflammation markers such as IL‐1β and TNFα using cortical RNA samples. There was no significant change on *IL‐1β* or *TNFα* mRNA levels in *Pen‐2* cKO cortices at 3 months compared with littermate controls (data not shown). In contrast, there was remarkable increase on mRNA levels for *IL‐1β* (Figure 4E) and *TNFα* (Figure 4F) in *Pen‐2* cKO cortices. Overall, the above results suggest striking inflammatory responses in *Pen‐2* cKO cortices.

### 3.5 Nonessential role of the Notch signaling in Pen‐2‐dependent neuronal survival

**Figure 5:**
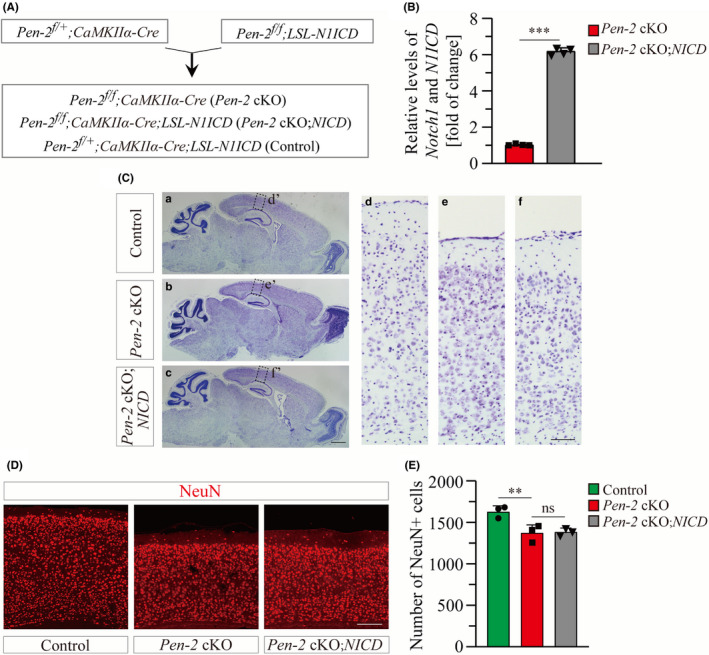
NICD does not ameliorate neuronal loss in *Pen‐2* cKO mice. A, Experimental plan for the generation of *Pen‐2* cKO mice expressing N1ICD conditionally in a Cre‐dependent manner. B, qPCR analysis on *Notch1 and N1ICD*. Relative mRNA levels of *Notch1 and N1ICD* were highly significantly increased in the cortex of *Pen‐2* cKO;*NICD* mice as compared to *Pen‐2* cKOs (***P < 0.001; n = 4 mice per group). C, Nissl staining. Brain sections of control, *Pen‐2* cKO, and *Pen‐2* cKO;*NICD* mice at 5‐7 mo were used. Note that there was decreased cortical thickness in *Pen‐2* cKO mice with and without NICD expression compared with controls. Boxed areas in (a‐c) were enlarged as (d‐f). Scale bar is 1 mm in (a‐c) or 100 μm in (d‐f). D, Representative images for fluorescence IHC on NeuN. Images were taken from the cortex. Note that there was decreased cortical thickness in *Pen‐2* cKO and *Pen‐2 cKO;NICD* mice compared with controls. Scale bar is 200 μm. E, Averaged number of NeuN^+^ cells in the cortex. There was no significant difference between *Pen‐2* cKO and *Pen‐2* cKO;*NICD* mice. There was significant difference between *Pen‐2* cKO and control mice (ns, not significant; **P < 0.01; n = 3 per group)

Notch is a major target of γ‐secretase.^17^ We previously demonstrated that expression of NICD rescues decreased population of neural stem cells caused by deletion of Pen‐2.^24^ To investigate whether the Notch signaling also plays a critical role in Pen‐2‐dependent neuronal survival, we crossed *Pen‐2* cKO mice to *N1ICD* transgenics to obtain *Pen‐2* cKO mice expressing NICD in Cre^+^ neurons in the adult cortex (Figure 5A).

First of all, qPCR analysis on total cortical RNA samples showed highly significant increase on *N1ICD* mRNA levels in *Pen‐2* cKO mice expressing NICD compared with *Pen‐2* cKOs without NICD expression (Figure 5B). Secondly, we examined brain morphology of mice at 5‐7 months by Nissl staining (Figure 5C), which revealed comparable thickness of the cortex between *Pen‐2* cKO mice with and without NICD expression. Thirdly, the number of NeuN^+^ cells did not differ between *Pen‐2* cKO mice expressing NICD and those without NICD expression (Figure 5D,E). It was much less in *Pen‐2* cKO mice with and without NICD expression than in controls (Figure 5D,E).

**Figure 6:**
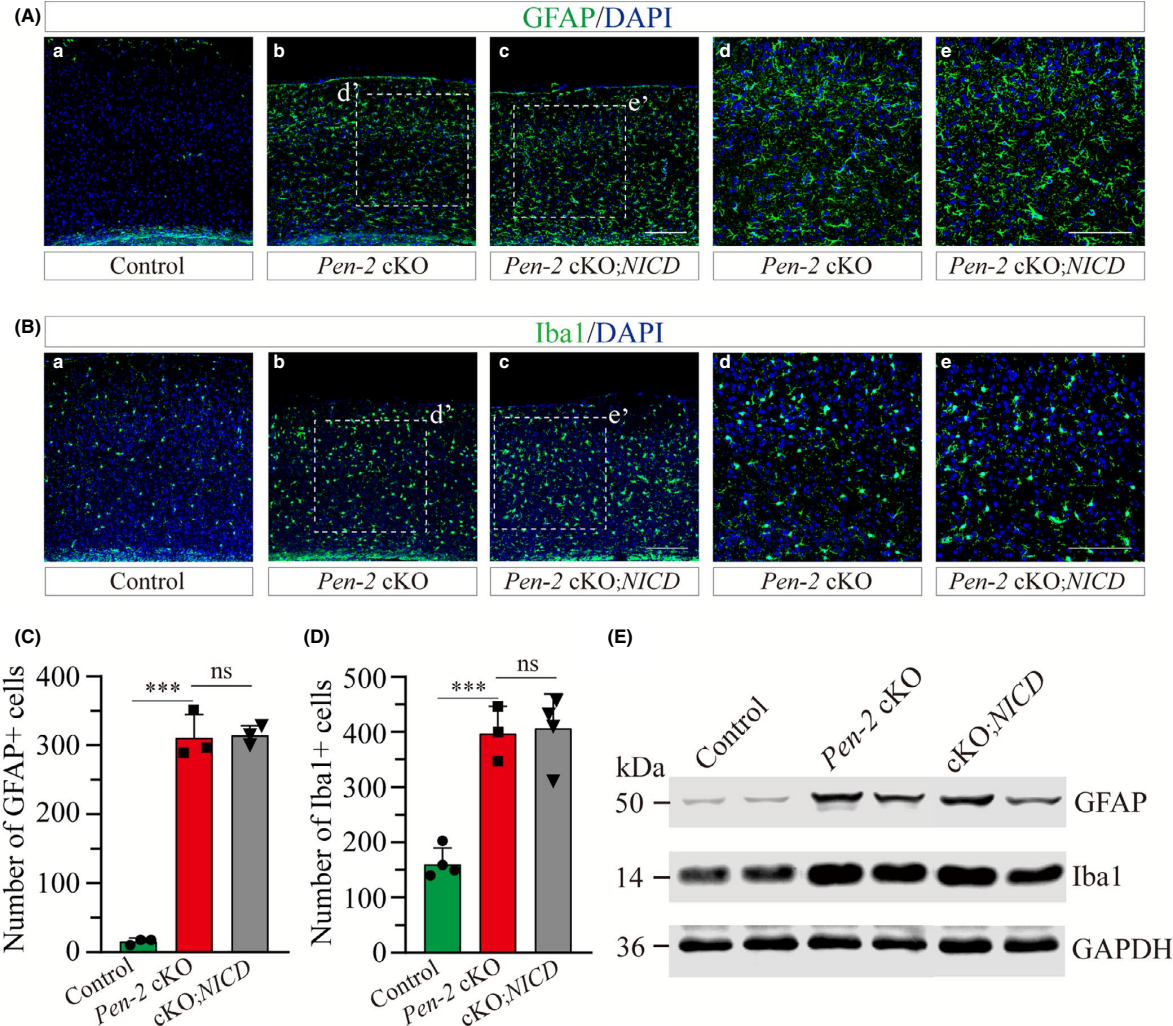
NICD does not reduce gliosis in *Pen‐2* cKO mice. A‐B, Representative images for fluorescence IHC on GFAP (A) and Iba1 (B). Images were taken from the cortex of control, *Pen‐2* cKO or *Pen‐2* cKO;*NICD* mice. Boxed areas in (b,c) were enlarged as (d,e). Scale bar is 200 μm in (a‐c) or 100 μm in (d‐e). C, Averaged number of GFAP^+^ cells in the cortex. There was no significant difference between *Pen‐2* cKO and *Pen‐2* cKO;*NICD* mice. There was significant difference between *Pen‐2* cKO and control mice (ns, not significant; ***P < 0.001; n = 3 mice per group). D, Averaged number of Iba1^+^ cells in the cortex. There was no significant difference between *Pen‐2* cKO and *Pen‐2* cKO;*NICD* mice. There was significant difference between *Pen‐2* cKO and control mice (ns, not significant; ***P < 0.001; n = 3‐4 per group). E, Western blotting on GFAP and Iba1. Cortical samples of control, *Pen‐2* cKO and *Pen‐2* cKO;*NICD* mice were used. Blots for GFAP or Iba1 were comparable between *Pen‐2* cKO and *Pen‐2* cKO;*NICD* mice

Moreover, we performed IHC on GFAP and Iba1 using brain sections from *Pen‐2* cKO mice expressing NICD (Figure 6A,B). While the number of GFAP^+^ cells in the cortex did not differ between *Pen‐2* cKO and *Pen‐2* cKO*;NICD* mice (Figure 6C), it was significantly increased in *Pen‐2* cKO with and without NICD expression compared with controls (Figure 6C). Similar results were obtained for Iba1 IHC. We did not find any difference on the number of Iba1^+^ cells in the cortex in *Pen‐2* cKO mice with and without NICD expression (Figure 6D). Finally, Western analyses confirmed no significant difference on protein levels of GFAP (cKO = 399 ± 17%; cKO*; NICD* = 413 ± 35%; n = 3‐4 mice per group; *P* > 0.6) or Iba1 (cKO = 183 ± 21%; cKO*; NICD* = 175 ± 18%; n = 3 per group; *P* > 0.6) between *Pen‐2* cKO mice with and without NICD expression (Figure 6E and Figure S4). Together, NICD rescued neither neurodegeneration nor neuroinflammation in *Pen‐2* cKO cortices.

## 4 DISCUSSION

Recent evidence has shown that Pen‐2 is implicated in familial AD.^25^, ^26^ Since global deletion of Pen‐2 causes early embryonic lethality in mice,^23^ this precludes the possibility to use *Pen‐2^−/−^* mice to study whether Pen‐2 plays a role in neuronal survival in the adult cortex. To overcome this problem, we employed the Cre‐LoxP techniques and generated viable forebrain neuron‐specific *Pen‐2* cKO mice (Figure 1). As expected, there is significant reduction on Pen‐2 expression in *Pen‐2* cKO cortices (Figure 1). Nicastrin is another essential subunit of γ‐secretase,^17^ and it exhibits significant decrease in *Pen‐2* cKO mice as well (Figure 1). We reason that deletion of Pen‐2 prevents the formation of the γ‐secretase complex so that the stability of other subunits is impaired in cortical neurons. In line with this view, forebrain neuron‐specific inactivation of presenilins or nicastrin causes decreased levels of Pen‐2.^37^ Moreover, previous in vitro studies have also shown that knockdown of one γ‐secretase subunit could significantly reduce protein levels of other subunits.^19^, ^38^ Overall, γ‐secretase subunits may depend on each other for stability in mature neurons.

While we have focused on Pen‐2 in this study, a number of recent studies have investigated the role of other γ‐secretase subunits in the adult cortex. First, it has been shown that conditional inactivation of presenilins causes memory loss, synaptic impairments, and age‐dependent cortical neurodegeneration.^39^, ^40^ Second, nicastrin was the second subunit identified to be critical for neuronal survival in the cortex.^37^, ^41^, ^42^, ^43^ Third, there is age‐related neuronal loss in forebrain neuron‐specific *Aph‐1* cKO mice.^44^ Together, the above evidence suggests that each γ‐secretase subunit may be equally important for the maintenance of mature neurons in the cortex.

Numerous mutations on *Psen1*, *Psen2*, *nicastrin,* and *Pen‐2* have been reported in familial AD,^8^, ^25^, ^26^, ^27^, ^45^, ^46^ but the relevant mechanisms are not clear. It has been proposed that loss of presenilin mechanism may play a key role in AD.^8^, ^9^, ^47^ However, since presenilins possess γ‐secretase‐dependent^17^ and γ‐secretase‐independent functions,^8^, ^48^ it remains unsolved which type of function may protect mature cortical neurons. Tremendous efforts have been made by independent groups to search for mechanisms underlying neuronal survival regulated by γ‐secretase subunits. First, Saura et al (2004) showed that presenilins may promote neuronal survival via Notch‐dependent activation of CREB‐binding protein (CBP). Since neuron‐specific inactivation of CBP does not cause age‐related neuronal loss,^49^ it is unlikely that CBP is involved in presenilins‐dependent neuronal survival. Second, apoptotic cell death has been reported in *nicastrin* cKO mice.^37^ In contrast, TUNEL^+^ cells are not detected in *Pen‐2* cKO cortices aged at 6 or 9 months (data not shown), suggesting that neuronal loss may not be caused by apoptosis. The discrepancy on TUNEL data for *nicastrin* cKO^37^ and *Pen‐2* cKO mice may be due to different *CaMKIIα‐Cre* lines used to generate these two different cKO mice. For example, the expression of Cre begins around P18 in *nicastrin* cKO mice^50^ but 6 weeks in *Pen‐2* cKOs.^30^

Molecular mechanisms underlying neurodegeneration caused by loss of γ‐secretase subunit are largely unclear. Since APP is one of the most important targets of γ‐secretase,^17^ recent studies have examined whether APP‐CTF, a cleavage product of APP by γ‐secretase, plays an important role in neuronal survival in the adult brain. Since it has been shown that accumulation of APP‐CTF is detrimental to neurons,^51^ Acx and colleagues (2017) generated *Aph‐1* cKO mice on the *APP* null background. However, there is no significant difference on the number of cortical neurons between *Aph‐1* cKO and *Aph‐1* cKO*;APP^−/−^* mice.^44^ Thus, deletion of APP and prevention of APP‐CTF do not ameliorate neurodegeneration in *Aph‐1* cKO mice. This finding excludes the possibility that neurodegeneration is due to accumulation of APP‐CTF. Although the Notch signaling is critical for the fate determination of NPCs,^52^, ^53^ the present study shows that conditional expression of NICD in forebrain neurons is insufficient to improve neurodegenerative phenotypes in *Pen‐2* cKO mice. Overall, neither APP‐CTF nor the Notch signaling serves as a key mediator for γ‐secretase subunit‐dependent neuronal survival in the adult cortex.

Reactive astrogliosis is a common response in injured brain associated with diseases such as trauma, infection, neurodegeneration, and ischemia.^54^ We observe significant astrogliosis but not microgliosis in *Pen‐2* cKO mice at 3 months of age, suggesting that astroglial activation occurs prior to microgliosis and neuronal loss in this neurodegenerative model. In line with this finding, previous studies showed early astrogliosis in *nicastrin* cKO mice at 3 months.^37^, ^42^, ^43^ Moreover, it has been shown that deletion of presenilins or Aph‐1 causes enhanced astrogliosis in the cortex.^39^, ^44^, ^55^ These results strongly suggest that inflammatory responses may play a pivotal role in neurodegeneration caused by loss of γ‐secretase subunit. Consistent with these findings, it has been proposed that uncontrolled inflammation drives the progression of neurodegeneration.^56^ The novel *Pen‐2* cKO model generated in this study may serve as an excellent tool to test the anti‐inflammation strategy for neurodegenerative diseases and to study mechanisms underlying neuronal maintenance dependent on normal γ‐secretase function. Answers to these questions may not only provide insights on the pathogenesis of AD but also promote identification of potential therapeutic targets.

## 5 CONCLUSIONS

Functional analysis on forebrain neuron‐specific *Pen‐2* cKO mice reveals that Pen‐2 plays an essential role in the survival of mature cortical neurons. Early astrogliosis takes place prior to the occurrence of evident neurodegeneration in *Pen‐2* cKO cortices, followed by severe astrogliosis and microgliosis. The failure on rescue of neurodegeneration and gliosis in *Pen‐2* cKO cortices by NICD suggests that the Notch signaling is not critical for Pen‐2‐dependent maintenance of neurons in the adult cortex.

## CONFLICT OF INTEREST

The authors declare no conflict of interest.

## Supporting information

Supplementary Material

## Data Availability

The data presented in this article are available from corresponding authors upon request (guyueym@njmu.edu.cn and chenguiquan@nju.edu.cn).
