# Supplementary material for: Neuron‐specific deletion of presenilin enhancer2 causes progressive astrogliosis and age‐related neurodegeneration in the cortex independent of the Notch signaling
Source: CNS Neurosci Ther. 2020 Sep 22;27(2):174–85. doi: 10.1111/cns.13454 (PMC7816208; doi:10.1111/cns.13454)
Supplement: Supplementary file 1 — Supplementary Material [file CNS-27-174-s001.pdf]

Full unedited gel/blot for Figure 1B

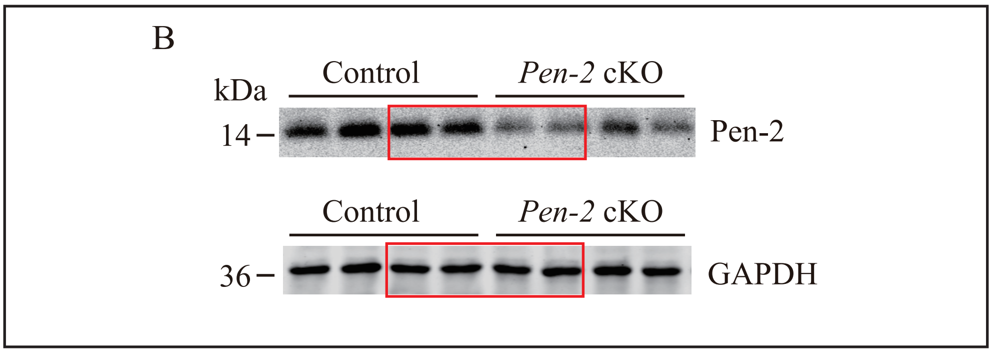

Full unedited gel/blot for Figure 1D

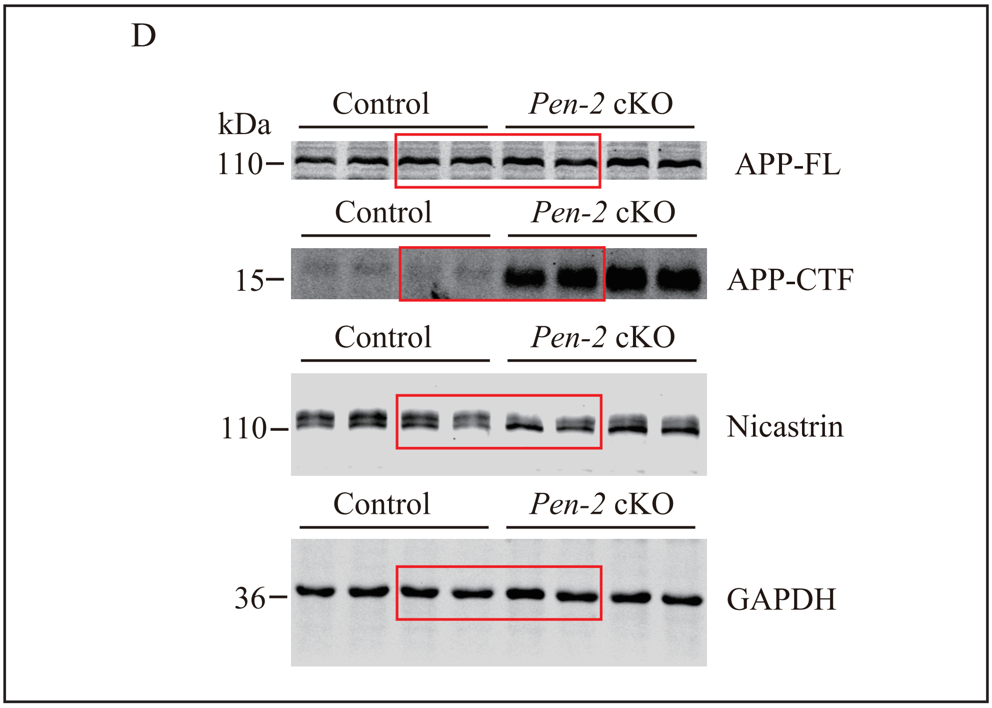

Supplementary Figure 1 Full unedited gel/blot for Figure 1.

Full unedited gel/blot for Figure 3C

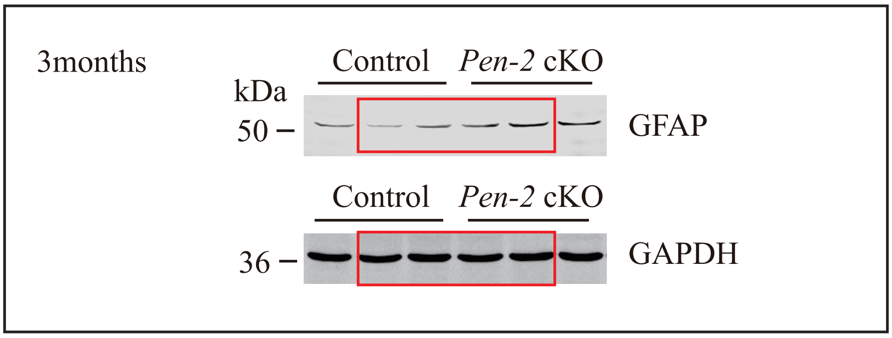

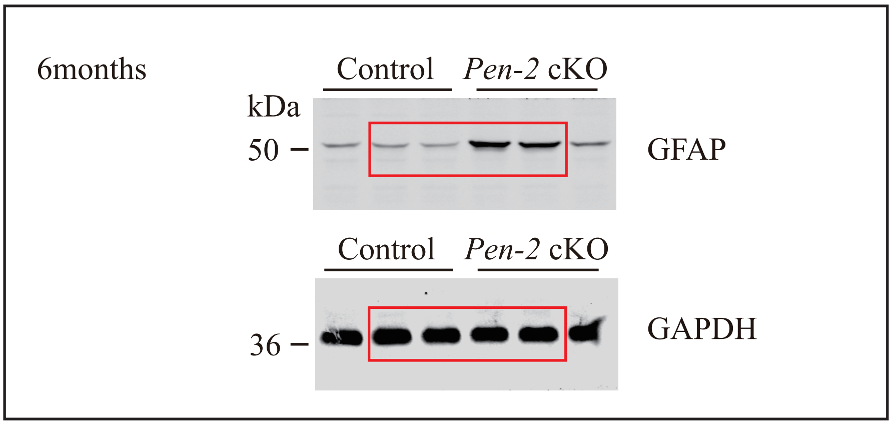

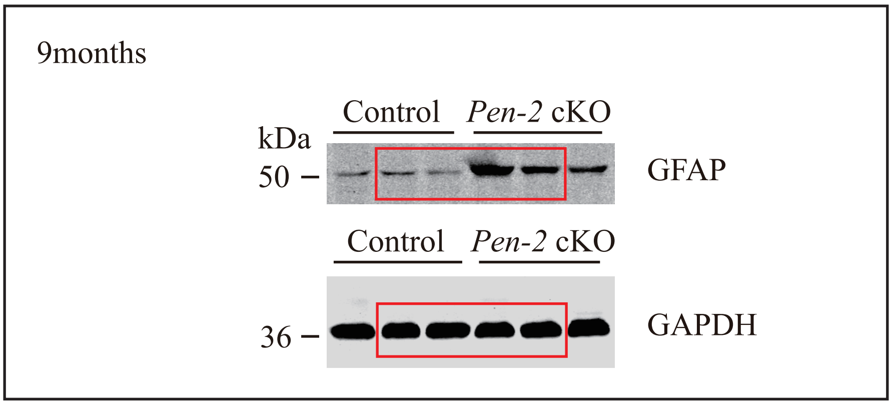

Supplementary Figure 2 Full unedited gel/blot for Figure 3.

Full unedited gel/blot for Figure 4C

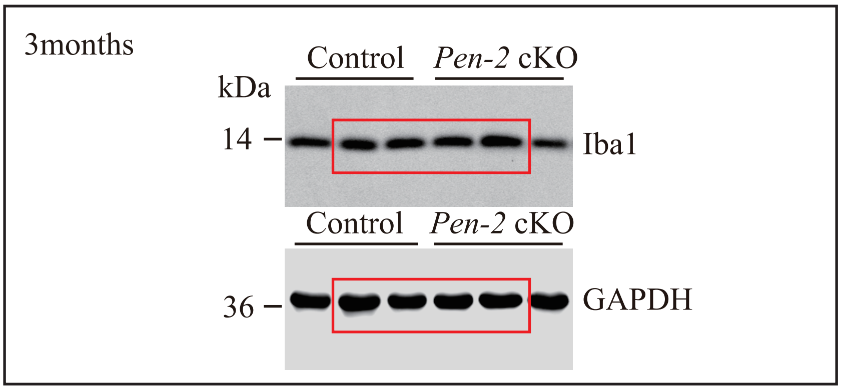

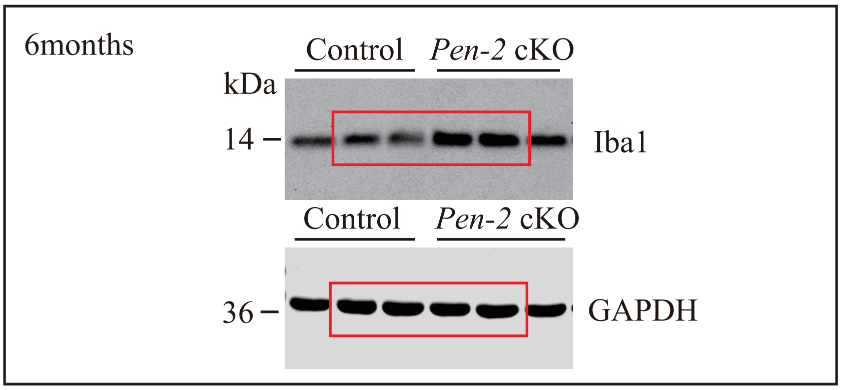

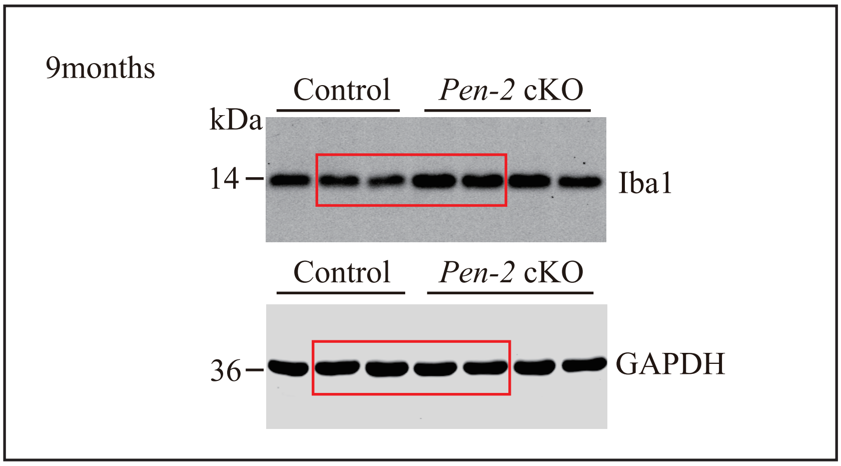

Supplementary Figure 3 Full unedited gel/blot for Figure 4.

Full unedited gel/blot for Figure 6E

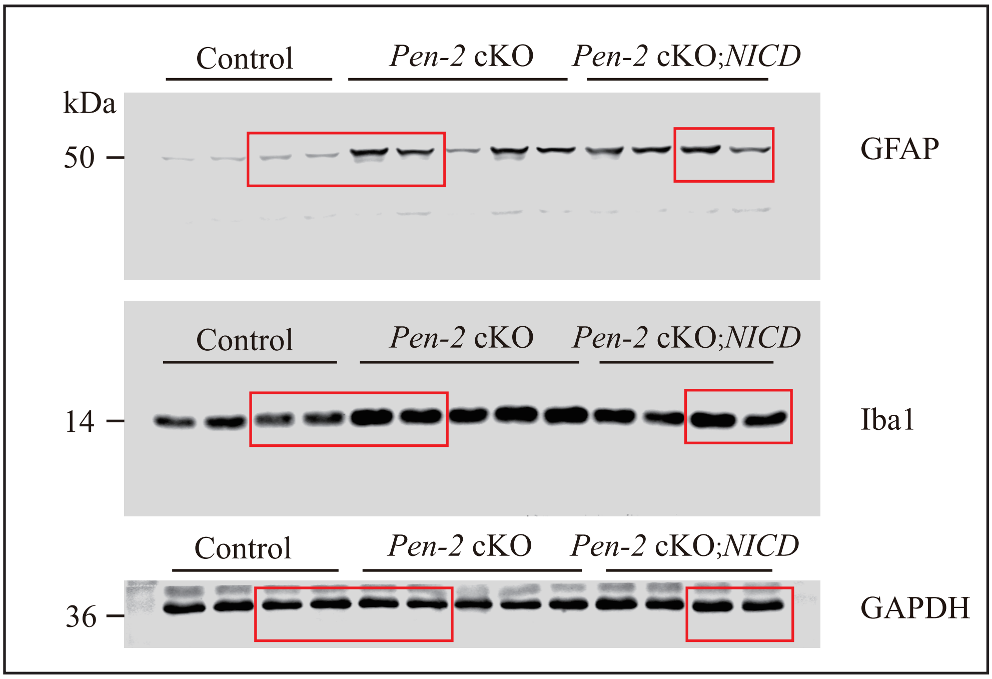

Supplementary Figure 4 Full unedited gel/blot for Figure 6.
